# Density Can Be Misleading for Low-Density Species: Benefits of Passive Acoustic Monitoring

**DOI:** 10.1371/journal.pone.0052542

**Published:** 2013-01-09

**Authors:** Tracey L. Rogers, Michaela B. Ciaglia, Holger Klinck, Colin Southwell

**Affiliations:** 1 Evolution & Ecology Research Centre, School of BEES, University of New South Wales, Sydney, New South Wales, Australia; 2 Cooperative Institute for Marine Resources Studies, Oregon State University and Pacific Marine Environmental Laboratory, National Oceanic and Atmospheric Administration, Hatfield Marine Science Center, Newport, Oregon, United States of America; 3 Australian Antarctic Division, Department of Sustainability, Environment, Water, Population and Communities, Channel Highway, Kingston, Tasmania, Australia; Utrecht University, The Netherlands

## Abstract

Climate-induced changes may be more substantial within the marine environment, where following ecological change is logistically difficult, and typically expensive. As marine animals tend to produce stereotyped, long-range signals, they are ideal for repeatable surveying. In this study we illustrate the potential for calling rates to be used as a tool for determining habitat quality by using an Antarctic pack-ice seal, the leopard seal, as a model.With an understanding of the vocal behavior of a species, their seasonal and diurnal patterns, sex and age-related differences, an underwater passive-acoustic survey conducted alongside a visual survey in an arc of 4,225 km across the Davis Sea, Eastern Antarctica, showed that while acoustic and visual surveys identified similar regions as having high densities, the acoustic surveys surprisingly identified the opposite regions as being ‘critical’ habitats. Density surveys of species that cannot be differentiated into population classes may be misleading because overall density can be a negative indicator of habitat quality.Under special circumstances acoustics can offer enormous advantage over traditional techniques and open up monitoring to regions that are remote, difficult and expensive to work within, no longer restricting long-term community assessment to resource-wealthy communities. As climatic change affects a broad range of organisms across geographic boundaries we propose that capitalizing on the significant advances in passive acoustic technology, alongside physical acoustics and population modeling, can help in addressing ecological questions more broadly.

## Introduction

Recent climatic change has affected a broad range of organisms with diverse geographical distributions. These include changes in phenology, the timing of seasonal activities of animals and plants, range shifts and changes in the distribution patterns of species, changes in the composition of and interactions within communities, and the structure and dynamics of ecosystems [1]. Range-restricted species such as those in polar and alpine environments are particularly vulnerable. They have shown severe range contractions and are the first groups from which entire species have gone extinct due to recent climate change [2].

So, where should we invest the limited conservation and research funds for protecting biodiversity [3]? Global conservation prioritization usually emphasizes areas with highest species richness or areas where many species are thought to be at imminent risk of extinction. However, such strategies may overlook areas where many species have biological traits which make them particularly sensitive to future human impact but are not yet threatened by them [4]; areas that are logistically difficult or expensive to work within; or areas governed by communities with fewer resources. Significant changes in physical and biological systems are occurring on all continents and in most oceans. However, the concentration of data available is predominantly on changes occurring in Europe and North America [5], where greater resources are available, and is biased towards changes occurring in terrestrial systems.

Recent studies have revealed that both abiotic changes and biological responses within the ocean, such as ocean circulation and chemistry, are substantially more complex than those occurring within terrestrial systems [6]. Also, synergistic effects between climate and other anthropogenic variables such as the exploitation of marine resources for example, particularly fishing pressure, are likely to exacerbate climate-induced changes within the marine system [6]. However, following change within the marine environment is substantially more difficult both logistically and financially.

Assessing occupancy-related metrics including measures of occurrence, density, abundance, habitat selection and range and distribution are fundamental requirements for effective research and wildlife management [7] as are meta-population studies and wildlife monitoring programs. Occupancy-related metrics are used broadly across a range of taxa as well as across ecological disciplines [7] but can be challenging and expensive to estimate for marine species that are rarely sighted. If the rarity of sightings is due to genuine scarcity, the need to extrapolate from a very small sample size to the entire population usually involves substantial uncertainty, and the use of the resulting estimates may be greatly limited by that uncertainty. On the other hand, if the rarity of sightings is due to the species' secretive behavior, the methodology must be robust to potential biases that could result from that behavior [8].

If individuals are undetected in a survey when they are actually present, referred to as a ‘false absence’, this will lead to underestimates of the true level of occupancy by the species being investigated [9]. The imperfect detection of a species can have serious consequences for habitat models even at modest levels [10], [11] and inferences about the ‘value’ of different habitats could be severely misleading [12]. Unfortunately, such challenges are faced in designing survey effort for estimating the abundance of many marine apex predators. As false absences can introduce large bias, obviously it is important that they are minimized [9].

For species difficult to survey via traditional visual techniques, either because they occur at low densities, or because they are cryptic, secretive, or just marine, traditional visual survey methods offer many challenges. However, for vocally active species the combined application of passive acoustics and spatially explicit models could offer an immediate and obvious benefit at a relatively modest cost as they can sample a much larger area for some marine species.

Acoustic surveying is often used to monitor terrestrial species which are secretive, elusive or uncommon, but exhibit species-specific, easily detectable vocalizations. Acoustic surveying is an indirect method of surveying, where the vocalizations of animals, rather than the animals themselves, are counted. As not all animals within the survey area are likely to be calling at the time of the survey, acoustic methods are often used to provide relative densities rather than absolute densities, where all animals in the area are detected. Auditory censusing has been used extensively in terrestrial studies of birds [13], frogs [14], and bats [15], and attention is now focused on the use of acoustic techniques for improving knowledge of site-occupancy for marine animals.

Marine animals are often difficult to spot and to distinguish at sea, even in the best conditions. As weather deteriorates sighting species becomes even more difficult. However, many difficult-to-survey marine species produce species-specific low-frequency stereotyped calls; which coupled with the extremely efficient propagation of low frequency sound through the ocean, sees acoustic techniques offering enormous potential for improving visual surveys [16], [17] and for use as stand-alone tools. Although limited to vocalizing animals, acoustic monitoring can often detect animals at greater distances than visual surveys and while the animals are underwater. The use of passive-acoustic techniques has tended to focus on the study of cetaceans where it has been used to improve estimates of: the probability of detection for visual surveys where the probability of detection is known or suspected to be low [16], [17], [18], [19], [20]; to study seasonal occurrences [21], distribution [22], and behavior [23], including dive patterns [24], [25].

The recent increase in the sophistication and capability of acoustic devices (reviewed in [26], [27]), has lead to the development of an array of systems from dipping units consisting of single or multiple hydrophones, acoustic tags which can be deployed on individual animals, multiple sensors on towed or bottom-mounted hydrophone arrays, to autonomous platforms which provide data across a range of spatial scales [27]. Additionally, passive-acoustic surveying has the advantage of being a robust data collection system where data collected is largely independent of collection error and inter-observer bias [28]. It enables data to be archived for future use providing useful information on multiple species not only the species targeted at the time of the study, and may be useful for monitoring long-term changes in community composition.

However, acoustic surveying methods are not without limitations. Distance sampling [29], the most commonly used method of estimating animal density and abundance, requires knowledge of the distance to calling individuals, and has been used to assess relative densities of sperm whales [30], dolphins [16], porpoises [31], and minke whales [32]. However, accurately measuring the distance to a calling animal underwater is not trivial and requires expensive, sophisticated equipment and/or processing of the acoustic data [20], [33], which may not be feasible in remote regions that are logistically difficult and/or expensive to work within. Under special conditions where distance sampling methods are compromised and the target species have highly stereotyped calling behavior, as is the case for some marine animals, conventional ‘timed-count’ methods, typically used for surveying songbirds [28], may be appropriate.

The approach to model sounds per animal over a unit of time, a timed-count, can be used to obtain an estimate of minimum population size (as a relative index) for species where there is information on the production of vocalizations (Acoustic behavior - including seasonal calling patterns, diurnal calling patterns, inter-individual stereotypy, inter-sexual stereotypy, audience effect and predictable calling rate over a unit of time), and the detection range of those vocalizations (Survey distance - empirical estimates and/or theoretical estimates calculated using call intensities). Quantifying the variability around vocal behavior coupled with simple modeling could provide an ideal cost-effective and repeatable surveying opportunity, under certain circumstances, for monitoring long-term community change within the marine environment. Passive-acoustics also has the potential to contribute additional information on population structure and habitat use when there is an understanding of the behavioral ecology of the species.

Here we used the leopard seal, *Hydrurga leptonyx*, as a case study to examine how passive-acoustics performed, as estimating its distribution patterns and abundance using traditional visual survey effort has faced challenges, with research hampered by the inaccessibility of the seals, as well as the logistical difficulties of conducting surveys within the Antarctic pack ice. Leopard seals are important top predators in the Antarctic ecosystem, and are a potential source of information on ecosystem interactions and environmental variability over a wide range of spatial and temporal scales. They are an ideal species to conduct an acoustic survey on as they are vocal, occur at low densities [8], their behavior makes them difficult to study and to survey visually as they spend long periods of time in the water making them unavailable to visual surveys. During a traditional visual survey conducted as part of an internationally coordinated program under the Scientific Committee of Antarctic Research (SCAR), the APIS (Antarctic Pack Ice Seal) program, there were so few leopard seals sighted that it was a major obstacle in developing population estimates from the data [8], [34]. The resulting range of plausible estimates were correspondingly very wide [8] and the authors cautioned the use of these estimates. Coupled with this high uncertainty are the peculiar logistical difficulties of working within the Antarctic pack ice, which made the visual survey effort expensive. Alongside one of the APIS programs' visual surveys [8] we conducted a passive-acoustic survey and here we propose to use this opportunity to examine how the passive-acoustic survey performed in comparison to the visual survey.

The leopard seal is an ideal target for a passive-acoustic survey as their acoustic behavior is highly stylized [35]. The acoustic behavior of the leopard seal is believed to be linked to their breeding behavior as it coincides with the timing of their breeding season, between November and the first week of January [36], and in captive seals with elevated reproductive hormones [36]. Adult male leopard seals have highly distinctive vocalizations with highly stereotyped calling rates [35], although sub-adult males have a higher calling rate than adults [37]. Male vocalizations are believed to function in mate attraction and/or territorial signaling as part of a long-range display [36].

Here we trial a timed-count survey as the logistical difficulties of working within the Antarctic pack ice meant that it was impractical at the time of the survey to identify the location of calling individuals over the large area that the survey was conducted. As leopard seals are known to call repeatedly in a stylized pattern for extended periods, of up to several hours per day during the breeding season, and frequent, predicable sound production is ideal for effective detection using passive-acoustic monitoring, they appear to be a good candidate for testing this form of passive-acoustic model. Here we explore whether passive-acoustics could improve our understanding of the spatial behavior of difficult-to-study species by identifying whether calling behavior can be used to identify important habitats at (i) the simplest level using the presence or absence of calling, (ii) by timed cue counts of calls using the variability around their vocal behavior coupled with simple models of survey range to quantify the relative densities of seals in different regions, and (iii) by using age-related information in calls to identify age-related distributional patterns. Encountering vocalizing female leopard seals is likely a rare event compared to calling males, and to date there has been no recordings made of known wild female leopard seals so our understanding of female calling behavior comes from captive animals only and these calls may not have been typical. Due to this the present study focuses on the calling behavior of male leopard seals alone.

## Results

### Calling Behavior to infer Spatial Patterns

**Figure 1:**
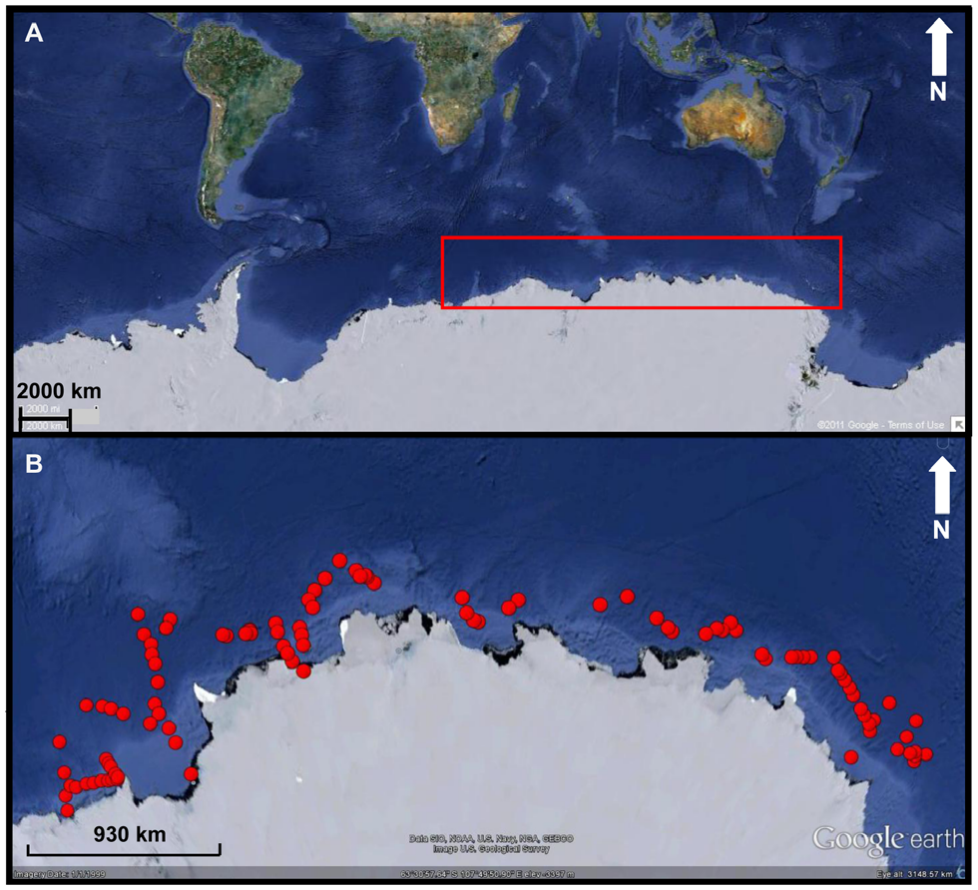
Sampling location site. (A) The red box identifies the area across the Davis Sea, Eastern Antarctica, over which the study was conducted; (B) A close up of the area identified in (A).The Red markers denote the positions of the 101 sites where 30-minute underwater passive-acoustic recordings were made from 4 December to 10 January of the following year within the pack ice between 64°31′S, 149°31′E and 67°17′S, 62°42′E within the Davis Sea, Eastern Antarctica. Maps courtesy of Google™ earth.

**Spatial behavior inferred using Numbers of Calls.** – To examine the calling patterns of male leopard seals over a larger spatial area, 30-min recordings were made in December and early January, at the height of the breeding season, at 101 sites distributed in an arc of 4,225 km across the Davis Sea (Figure 1B). A total of 38,270 leopard seal calls were counted from the 101 recording sites across the Davis Sea. The L call was the dominant call (54%; 20,768 calls, mean = 185±131_s.d._ calls/site) acoustically detected at most (96%, 97 of 101) of the sites. At the four (4) sites where no L calls were heard none of the other leopard seal calls (H, M, D, O) were heard. At the sites where L calls were detected, the H call made up 19% of all calls (7,434 calls, mean = 66±71_s.d._ calls/site), the D call 13% (4,901, mean = 44±39_s.d._ calls/site), the O call 8% (2,994, mean = 27±28_s.d._ calls/site), and the M call 6% (2,173, mean = 19±23_s.d._ calls/site). At any one site several overlapping calls could be heard in both the near and far fields indicating that two or more seals were calling at any one location.

**Figure 2:**
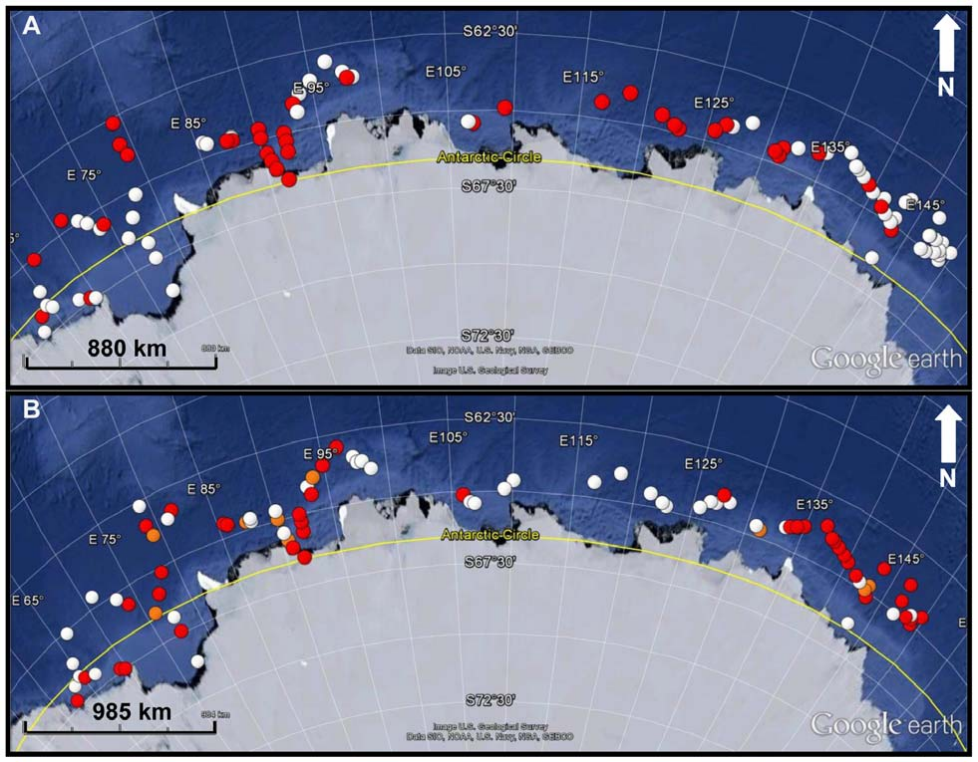
Spatial distribution of seals. The spatial pattern of leopard seals as inferred from the calling patterns in 30-minute underwater acoustic recordings made at 101 sites within the pack ice between 64°31′S, 149°31′E and 67°17′S, 62°42′E within the Davis Sea, Eastern Antarctica (Figure 1A) from 4 December to 10 January of the following year: (A) Density of seals: white = <2 seals/km^2^; red = >2 seals/km^2^. (B) Age Class: white = more sub-adult than adult calls counted; orange = equal number of adult and sub-adult calls; and red = the presence of more adult calls than sub-adult calls; all maps courtesy of Google™earth.

Although seals were distributed across most (96%) of the 101 sites across the Davis Sea (Figure 1B) there were regions where higher rates of leopard seal calls were detected as identified in Figure 2A. Calculating density estimates requires an understanding of the survey range and detection probability but in this study it was not possible to measure either. The survey range for each site was estimated using empirical models which were likely to be biased (see Materials and Methods). As an initial approach we used the number of L calls alone as an occupancy metric as it excluded the influence of both survey area and detection probability. The number of L calls/30 min was proposed for use as the L call was the dominant call, and was produced by the seals in a stereotyped fashion.

Seals were found to be distributed differently depending on age (age-class was determined by the fundamental frequency of the calls), with younger, sub-adult seals found in sites where significantly higher numbers of L calls (ANOVA: F_(2, 97)_ = 10.23; p = 0.00009) were heard, whereas adult seals were detected in sites with a lower number of L calls. Sites with both adult and sub-adults detected had intermediate calling rates (Figure 2B).

**Spatial behavior inferred using the Relative Density of Seals.** - A density (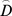) of 5,926 (CV = 12%; 95%±CI = 4,552 to 7,301) leopard seals was estimated for the total area of 19,820 km^2^, which equates to 0.31 seals/km^2^. Regions with higher densities of leopard seals are identified in Figure 2A.

The density of seals predicted was influenced (Multiple Regression: R^2^ = 0.38; d.f. = 3,97; p = 0.0000001) by the level of the background noise (b* = 0.88) present at a given site as masking will reduce our ability to detect the animals, as well as by the area surveyed (b = 0.37), but was not influenced by the environmental variable, the pack ice cover (b = −0.11).

**Figure 3:**
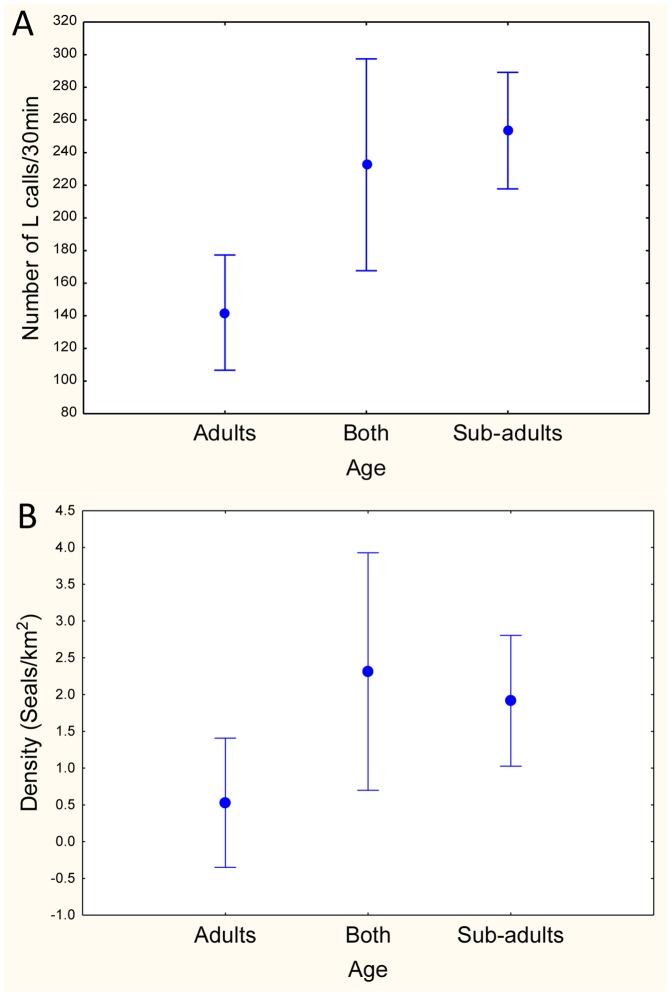
Influence of age class. Plots of the mean and 95% confidence intervals of the influence of age-class on: (A) the number of L calls in 30-minute underwater acoustic recordings; and (B) the density of seals (seals/km^2^) predicted from the number of calls at each recording site within the Davis Sea pack ice (n = 101). Adults = mostly adults; Both = adults and sub-adults; and Sub-adults = mostly sub-adults.

**Age-related distribution.** – The seals were distributed differently depending on age, with adult seals tending to be in areas of significantly (ANOVA: F_(2, 97)_ = 3.22, p = .044) lower seal densities, whereas younger seals, the sub-adults, tended to be in areas with higher densities (Figures 2, 3). In regions identified acoustically as having both adult and sub-adults individual's present, densities were found to fall between areas solely of adult, sub-adult seals or both adult and sub-adult seals.

## Discussion

### Calling Behavior to Infer Spatial Behavior

Here we capitalized on the leopard seals calling behavior in both the stereotypy in the rate at which they produce the most frequently heard L call, as well as the potential to use this same call to identify different age cohorts. Passive acoustics provided the ability to distinguish between the calls of adults and sub-adults using their acoustic features namely the fundamental frequency (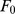) of the L calls, the rate of vibration of the vocal folds [37], [38], as an age-related classification tool as older seals produce calls with higher fundamental frequencies [39].

Wide-spread belief is that across vertebrates lower-pitched vocalizations are typically associated with larger and/or higher quality males and so the calls produced by larger, dominant males in inter- and intra-sexually selected displays will have lower fundamental frequencies [40]. This is the case for many taxa, including frogs [41], several non-human primates [42], [43], and the Amazonian manatee [38], but it is not universal, and is not the case for the leopard seal [39], nor the red deer [40], [44]. In red deer, altering the subglottal pressure can increase the fundamental frequency of the call especially in deer with strong chest muscles and higher lung capacity and those that roar more frequently [45]. Female red deer have been shown to preferentially select males that produce higher, rather than lower ‘pitched’ roars [40], [44]. So for the leopard seal the higher fundamental frequencies of more mature animals may similarly, be related to physical characteristics.

Fundamental frequency does not always provide information on size, and in the case of the red deer, formants are more reliable in conveying information about the caller [45]. We selected the fundamental frequency as a classification trait as it is highly conserved in recordings with poor signal-to-noise ratios, where formants are likely to be lost. So, from an acoustic survey perspective it is a valuable tool if it is capable of identifying potential age-related differences.

The continuous, consistent, stylized calling we report here is common among the acoustic displays of marine animals distributed at low densities and is likely an important fitness display, however, this stereotypy also provides researchers and managers with an ideal tool for undertaking occupancy studies. With an understanding of the change in call features over the lifespan of an individual, and how calling rates change through a season, we examined the occurrence of adults and sub-adults, which may reflect habitat ‘quality’ over large spatial regions by examining the patterns of presence of adult and sub-adult callers.

As leopard seal calls were detected acoustically in most of the study sites, in this instance using passive acoustics to identify the simple presence or absence of animals as a survey tool offered less value. If we had intended to use acoustics as a spatial ‘presence or absence’ detection method we would have identified nearly all areas as being important habitat, with no ability to distinguish between locations. As solitary leopard seals call during the breeding season as part of a long-range display, their calls are designed to travel great distances underwater. This means that the acoustic range is broad and therefore not surprising that the likelihood of detecting animals at any of the sites was very high, nor that at any site there were several overlapping calls in both the near and far fields, indicating that two or more seals were detected at any one location.

The acoustic survey model predicted a high mean relative density of seals (0.31 male seals/km^2^). This is a conservative estimate as it only monitored a proportion of the population, here the calling male leopard seals. However, there are significant limitations to our acoustic survey approach as we have not considered the probability of detecting a cue (a calling animal) within the survey area. Most density estimation methods are based on estimates of the probability of detecting calls as a function of distance [46]. This was not possible in this study as we had a single sensor. However, a recent alternate approach estimates the probability of detecting calls from single sensors by coupling field simulations of animal sounds and modeling [46] which would have been ideal in this circumstance but was not conducted at the time of the study. The use of directional frequency analysis and recording (DIFAR) sonobuoys in the future could be used to improve the localization of vocalizing animals. In addition, here we use the sonar equation with spherical spreading to predict survey area. This is only true when the sound energy is free to spread in all directions, which only occurs at close ranges or in deep oceans. For simplicity we used spherical spreading to model propagation as it provides results that are highly averaged over time, range and depth, so is a useful general test of potential operating range.

At the time of our acoustic survey, and from the same survey platform, the ice breaker the *RSV Aurora Australis*, a dedicated visual survey, which included both visual ship-based and aerial observations, was conducted as part of the SCAR APIS program. Compared to our study the visual survey estimated a substantially lower density of leopard seals (0.006 leopard seals/km^2^ [8]) for the same region and time. In the 3,083 km of ship transects in the visual survey a total of 17 leopard seals were seen, 14 of which were within 400 m of the ship. At a distance of 400 m from the ship detect-ability is estimated to be close to perfect, provided that animals are available for detection that is that they are hauled out on the ice (Southwell, unpublished data). Earlier reported estimates of leopard seal densities obtained by traditional visual surveying have ranged between 0.003 and 0.151 leopard seals/km^2^ [8], [47]–[60]. By comparison, the results of the present study are high, particularly considering they are a conservative estimate as they are not likely to account for the female seals.

For the Antarctic pack ice seals, the visual and acoustic surveys operate independently of one another, with each measuring potentially different individuals. The visual survey encounters animals hauled out on the ice while the acoustic survey encounters animals calling underwater. The two survey techniques are largely detecting complementary (in the mathematical sense) populations. Visual surveys predominantly detect hauled-out animals that are necessarily not calling and thus undetectable by acoustic surveys. Acoustic surveys detect submerged calling animals which are undetectable by visual surveys. An implication of this is that these survey modalities are not “independent” in the technical statistics sense of the word. There is almost zero probability of an animal being detected by both surveys even though either survey modality has non-zero marginal probability of detection.

As seals are available to visual surveys only when they are on the ice, correction factors were developed to account for the seals in the water and therefore unavailable to the APIS visual survey [8]. The population sampled during the visual survey may have been biased towards hauled out females as males may haul out less often than the females [35].

The timing of the visual surveys, during the austral spring/summer, coincides with the leopard seals breeding season [35]. During this period females need to haul out onto ice floes to give birth to their pups, at which time they are available to sighting surveys, at least during the time of nursing. While still unknown at this time, estimates of the length of the lactation period for the leopard seal vary from 10 days to 8 weeks [61]. It is not known whether breeding females remain continuously on the ice for the duration of the lactation period, as do other pack ice seals such as the crabeater seal, or whether they are like Weddell seals and only remain on the ice intermittently [62]. It is also unclear whether individual breeding females haul out across the entire breeding season or, because of asynchrony in pupping, for just a portion of the season. Substantial asynchrony in births, as suspected [61], would mean that only a low proportion of breeding females would be on the ice with a pup at any one time. In addition, the continuous lighting regimes of summer and winter at high latitudes may have caused the loss in daily rhythmic activity, as seen in the Arctic reindeer, and it has been proposed that this absence of circadian rhythmicity may be ubiquitous in polar vertebrates [63]. If individuals in a population vary substantially in their behavioral patterns, here the tendency to haul out, and so vary in their availability to a visual survey, using a generic correction factor that does not reflect the diversity that exists would bias the results of a visual survey.

During the summer breeding season males invest heavily in calling. From the bottom-mounted buoy data males were calling underwater throughout the day during the period of the visual and acoustic surveys. Using the correction factor based on the haul-out behavior of females to account for the time period that seals were not available to the visual survey, would over-estimate the proportion of time that the male population spends on the ice, and therefore under-estimate overall leopard seal abundance.

In the visual surveys, there were large numbers of zero (absent) observations for leopard seal sightings across the area surveyed [8] which is at odds with the extremely high occurrence of leopard seal detections in the same region from the coincident passive acoustic survey. This suggests that either seals were there but were not detected by the visual survey, reflecting a false absence record, and/or that a larger area was surveyed acoustically. The area surveyed acoustically was in fact larger covering around 19,820 km^2^, whereas the visual sampling from the same survey platform covered 3,083 km^2^. This broader survey range may explain the surprisingly low CV for the acoustic estimates. In this instance the passive acoustic recordings sampled a much larger area over a fixed ship time and minimized the high zero data counts, making them a more effective use of the expensive survey platform. For vocal, low density species like the leopard seal acoustics offers great advantages.

The visual survey approach [8] was not robust to false absences, that is, where one or more leopard seals were present in a segment and amenable to survey but no presence was recorded. A false absence will lead to underestimates of the true level of occupancy [9]. The imperfect detection of a species also has serious consequences for habitat models. False absences can cause estimates of habitat effects to be biased, even at modest levels, particularly if detection probability varies between habitats [10], [11]. The male seal's calling behavior could be causing false absences, thereby compromising the visual surveys further. Inferences about the ‘value’ of different habitats could be severely misleading if detection probabilities are correlated with occupancy probabilities [11], [12].

The acoustic data identified similar regions to the visual surveys [8] as having higher densities of leopard seals. However, the acoustic surveys identified the higher-density areas as having more sub-adult seals where as the lower-density areas had adult seals. Sub-adult leopard seals tend to be at higher densities compared to adult seals [64]. This supports the concern that density can be a negative indicator of habitat quality for some species [65]. High density as an indicator of ‘high’ quality habitat can be misleading in species where dominant individuals secure space in prime habitats, forcing subordinate individuals to aggregate in large numbers in marginal areas [65], [66]. If we assume dominant animals claim larger areas this may result in the crowding of less-fit animals into less desirable habitats in a two-tiered dominance system: strong individuals claiming roughly comparable areas with lesser individuals' sharing the remaining habitat. This would mean that the more dense populations would be largely composed of sub-adults with fluid and not well-established dominance hierarchies. Another plausible model however, would be a multi-tier hierarchy, where the most fit individuals claim large areas, slightly less fit animals are still able to defend less desirable smaller areas, and so on through several levels until only the pool of least fit animals are unable to defend any area at all. This may explain the distribution pattern we see from the acoustic data which showed regions not only of mostly adults or mostly sub-adults but also areas intermediate between these, equal in both adults and sub-adults.

Unlike the acoustic surveys, in this circumstance visual surveys did not have the capacity to provide age-related information. Density surveys that cannot differentiate between population classes could be misleading for species where overall density can be a negative indicator of habitat quality because dominant individuals secure prime habitats.

A challenge to traditionally used visual surveying methodology in the marine environment is the potential biases that result when the target species is difficult to visually survey, either because they are found at low densities, or because they are cryptic, and/or secretive. Traditional visual survey methods also offer challenges for vocally active species which vocalize or employ bio-sonar to feed underwater. Unfortunately, this is the case for many marine species. The combined application of passive acoustics and spatially explicit models can offer an immediate and obvious benefit when there is an understanding of the acoustic behavior of the target species. Passive acoustic mechanisms offer ways of sampling large areas of the ocean over long time periods and at a relatively low cost. When designing an acoustic survey it is important to consider the acoustic behavior of the target species and where a species is known to alter its acoustic behavior, such as by season or time of day, surveys need to be conducted at standard times, or correction factors applied. The results of this study indicate that Antarctic pack ice seals such as the leopard seal are well suited to acoustics surveys. In fact, visual surveys alone are likely to vastly underestimate the population of leopard seals, and are misleading in identifying regions of important habitat. As leopard seal densities are likely higher than previously thought, and since abundance estimates are extrapolated from density calculations [8], it follows that leopard seal abundance may also be significantly higher than current figures suggest.

## Materials and Methods

### Calling Behavior to Infer Spatial Patterns

**Data Collection.** - Underwater passive-acoustic recordings were made from 4 December 1999 to 10 January 2000 at 101 sites within the pack ice between 64°31′S, 149°31′E and 67°17′S, 62°42′E within the Davis Sea, Eastern Antarctica (Figure 1). This timing coincides with the peak underwater vocalizing period for the leopard seal and the height of their breeding season. At the time of the survey this comprised an area of 1,500,000 km^2^ and included all areas with >^1^/_10_ ice-cover between 64°E and 150°E [8]. This area would represent the majority, if not all, of the breeding population between these longitudinal boundaries [8]. The surveys were conducted as a series of points along a cruise track line. While the sampling regime was random along this cruise track its path had been pre-designed specifically for visual rather than acoustic surveys.

At the time of the acoustic surveys a visual survey for pack ice seals was being conducted off the same survey platform, the *RSV Aurora Australis* [8]. Each underwater recording was made remotely using a sonobuoy (Sparton Electronics AN/SSQ-57A) which sampled over a frequency range from 10 to 22,000 Hz. The position of each recording was recorded using GPS as the sonobuoy was deployed. The omni-directional hydrophone from each sonobuoy was lowered to a depth of 18 m below the water's surface. Signals were received back on the survey platform using two, 9-element custom-built stainless steel Yaggi antennas (YH09, RF Industries Pty Ltd) with a series of AR2001 receivers (AOR Ltd, AR 2001). The antennas were secured to the ship's mast at a height of 30 m above sea level. The signal was recorded using a Sony Digital Audio Tape recorder (DAT TCD-D8) with a frequency bandwidth range between 10 and 22, 000 Hz±3 dB. Recordings of at least thirty-minute duration were made at each acoustic survey point between 1600 and 0300 hours (local time), coinciding with what was believed to be the diurnal calling behavior for the leopard seal [67].

Leopard seals off the Eastern Antarctic produce five species-specific call types [73], [69]: the Low descending trill (D); the Hoot with low single trill (O); the Medium single trill (M); the High double trill (H); and the Low double trill (L) (Table 1) as part of a long-range stereotyped display. At each of the 101 recording sites across the Davis Sea the number of each type of leopard seal call (D, O, M, H, L) within the 30-minute recording were counted by a manual observer using Spectrogram Version 16.0 (Visualization Software LLC).

**Table 1:** Acoustic characteristics of leopard seal calls.

Variable	Mean ± s.d. (n)
**Low descending trill (D)**	
Min frequency (Hz)	311±10*
Frequency band (Hz)	200–1250
**Hoot with a single trill (O)**	
Min frequency (Hz)	195±21*
Frequency band (Hz)	200–250
**Medium single trill (M)**	
Min frequency (Hz)	1552±164*
Frequency band (Hz)	1600–2000
**High double trill (H)**	
Adult Min Freq (Hz)	2711±66.3 (21)
Sub-adult Min Freq (Hz)	2660±63.2 (12)
Frequency band (Hz)	2500–4000
**Low double trill (L)**	
Adult Min Freq (Hz)	321±7.2 (28)
Sub-adult Min Freq (Hz)	309±6.7 (23)
Frequency band (Hz)	250–630

The mean ± Standard deviation (s.d.) of the acoustic characteristics of the leopard seals underwater calls from Rogers [39] and Rogers et al. [35], [73] Min frequency (Hz) – represents the minimum frequency measured from the calls; Frequency band (Hz) - represents the frequency band used to measure the SL and corresponds to the −3 dB points on either side of the peak frequency for each of the call types.

* denotes that measurements were not made in this study but were taken from Rogers [39] and Rogers et al. [73].

In order to convert sounds into animal numbers, it is necessary to either locate where each different sound is made, that is to identify the location of each calling animal, or to calibrate the number of sounds detected with independent data on the number of sounds made per animal over a unit time (timed cue count). Here the survey was conducted as a timed cue count as incorporating distance measures into sampling was not possible. In order to estimate the abundance of a species using underwater acoustic recordings we need to take into account not only the acoustic behavior of the animal, that is the stereotypy of their calls and temporal pattern of calling behavior, but also the detection probability of the animal's calls themselves, which accounts for the physical acoustics of the call as well as the features of the underwater environment.

We used components of the equation described by Marques et al. [68] to estimate a relative density:
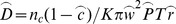
Where:


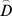
    is the estimated density,


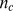
    is the number of detected cues,


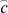
    is the estimated proportion of false positive detections,


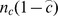
 corresponds to the number of detected cues that were actually from the target species,


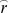
 is the estimated cue production rate.


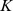
 is the number of replicate sensors used,


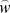
 is the distance away from the hydrophones beyond which cues are assumed to not be detected,


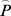
 is the estimated average probability of detecting a cue made within distance *w*,


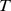
 is the time of recording, and

We do not consider here (1) the estimated average probability of detecting a cue made within distance w (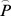) because it was not possible to get empirical measurements of the distance to callers; or (2) the estimated proportion of false positive detections (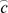) as we used manual detection rather than automated detectors.

**Number of cues detected (** 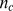 **) and Time of recording (** 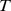 **).** - The number of leopard seal L calls within a 30-minute period at each site was counted by a manual observer using Signal 3.1 (Engineering Design, Belmont, USA) and SpectraPRO 3.32 (Sound Technology Inc., USA) so that the time of the recording at each site (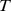) was 30-minutes. The assumption was made that the vocalizing animals did not change position or stop calling throughout the sampling period which may not always be the case. A period of 30-minutes was selected as the seals' stereotyped calling sequences are comprised of a two-minute calling period interspersed with a one to one and a half minute non-calling period (Figure 2 [39]). By selecting a 30-minute recording period we incorporated fifteen (15) two-minute periods of resting behavior (silent periods) and fifteen (15) two-minute periods of calling behavior. Over a 30-minute period we had the likelihood of recording 32 L calls per leopard seal [69], [39]

**Cue production rate (** 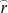 **).** - A fundamental assumption underlying an acoustic survey is that the target species have distinctive species-specific call(s). To use cue counting there needs to be an understanding of the cue-production rate per animal to convert estimates of cue density to animal density. Cue-counting involves counting for a known period of time the number of detected acoustic cues produced by the animals of interest, and appropriately scaling this number of detected cues to estimate animal density [68]. We used species-specific information on the degree of variability around cue production rates of the leopard seals Low double trill call of 30.8±7.4 calls in 30 minutes [35] to derive a cue production rate (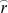) of 1.03 calls per minute.

**Number of replicate sensors used in the Survey (** 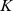 **).** - 101 fixed-point underwater passive-acoustic recordings.

**Distance from the hydrophone beyond which cues are assumed to be undetected (** 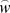 **).** - The detection threshold distance of the L call was determined using the equation for spherical spreading to calculate the distance over which a call would travel before transmission loss reached the value predicted by the sonar equation, this is the detection threshold distance (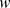) where the signal-to-noise ratio (SNR) is assumed to be one so that:
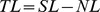
Where:


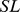
 is the on-axis source level of the sound source,


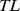
 is the range-dependent transmission loss,


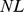
 is the spectral noise level of any masking noise at the receiver position.

Spherical spreading is only valid in an unbounded medium with constant sound speed. Such theoretical conditions rarely exist in real oceans, especially over long distances where the presence of ocean boundaries, refraction and scattering results in the need for complicated models of TL [70]. Sound propagates by spherical spreading when the sound energy is free to spread in all directions, which occurs at close ranges and in deep oceans. Here TL was considered to be due to spherical spreading losses because at the frequency range of the leopard seals Low double trill, between 250 and 315 Hz, absorption loss is low, the study was conducted in deep water (3000 m) where there were few boundary surfaces, and detections were made over relatively short distances.

Under these circumstances the spherical spreading law is a reasonable first approach in assessing the performance of passive-acoustics, but is not ideal and is likely to be underestimating the detection radius. At the time of the survey, through the austral summer, the Davis Sea tends to stratify to form a summer surface layer, the thickness of which depends upon the local sea-surface conditions. The recording locations were made in regions where the summer surface water was likely to be at a maximum between 30 to 40 m thick [71] and the hydrophone is likely to have been within this layer as are the seals as they are short shallow divers (Rogers, unpublished data). The survey sites were located out from the coast through the Davis Sea, incorporating Prydz Bay, on the seaward side of the shelf (1000 m isobath) in waters with a depth of 3000 m (Figure 1B).

The detect-ability of a call will depend on the signal-to-noise ratio; which is the interplay between the 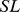 of the call, background 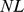 and 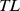. As a call travels farther away from the source the intensity of the received signal diminishes to a point at which the human ear or signal processing equipment can no longer pick out the signal from the surrounding background noise. This point is known as the detection threshold, and the distance at which it occurs is the detection threshold distance (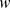). This assumes that the detect-ability threshold to which an observer can detect the call will be at a signal-to-noise ratio of 1 (0 dB) which was not likely the case. The L call is a highly stereotyped, coherent pulsed call (chirp/sweep) type which is likely to be detected by an observer below a signal-to-noise ration of 1 (0 dB) if the background noise is incoherent, due to gain from pulse compression. The consequence of this is that we are underestimating the detection range for L calls.

The probability of detecting a vocalization depends on the acoustic conditions of the surrounding environment. To determine the distance to which cues could be detected, the survey area of each buoy, we modeled the range of the L call using empirical measurements of (i) source level (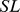) mean 169.3 dB re 1 µPa rms at 1 m [72]; (ii) background noise (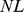); (iii) transmission loss (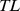), and (iv) the acoustic detection threshold distance (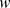).

**Underwater background noise level (** 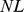 **).** - As ambient noise levels mask the animals underwater sound reception it is essential to measure the ambient noise level *in situ* at the frequencies of interest to predict its impact on call transmission at any particular site. The underwater background noise level (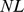) was calculated over the frequency band of the L call (Table 1) at each survey point (the point of deployment of for each sonobuoy) within the pack ice. The sound pressure level of underwater background noise is not a pure sound of only one frequency but a combination of sounds which includes various frequency components. To account for the contribution made by different frequencies we analyzed the sound pressure levels within ^1^/_3_ octave bands over the frequency band representative of each of the call types (Table 1) using a spectrum analyzer (SpectraPLUS 2.32, Sound Technology Inc., USA). Recordings at each location were of 30-minute duration, long enough for noise levels to have changed, particularly given that the ship, a likely contributor to the background noise, was moving away from the sonobuoy during the recording. To account for possible variability, an average voltage output (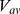) was calculated at each site by averaging the voltage output measured from six equidistant 4-second clips at each site at time periods: 0, 5, 10, 15, 20 and 25 minutes from the commencement of the recording. The 4-second samples were taken where seals' were not calling to ensure that the sample was representative of underwater background noise levels rather than the seals' calls. However, in a few circumstances where there was a high amount of calling this was not always possible.

**Transmission Loss (** 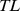 **).** - Transmission loss is the accumulated decrease in acoustic intensity as an acoustic pressure wave, here the seals' call, propagates outwards from the source of the sound, here from the seal. Values of 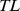 are determined by the rate of spreading of the radiated sound, by absorption and scattering losses at the sea surface and ocean bottom, and by absorption within the water column. However, an estimate of 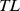 can be obtained from a simple determination of the spreading loss due to spherical spreading:
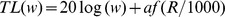
Where range (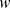) is the detection threshold distance, the distance from the sound source, expressed in meters (m), and 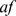 is the frequency dependent absorption coefficient in dB km^−1^. 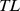 was modeled using the spherical spreading law.

**Accounting for loss and gain of signal due to the system.** - Within a recording and receiving system are a series of components that individually influence the gain. In order to calculate the sound pressure level we considered the contribution of each component within the system to the gain (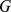).

To account for the contribution made by the different frequencies we analyzed the sound pressure level (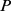) within ^1^/_3_ octave band levels. The sound pressure level was calculated as:
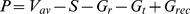
Where:


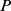
 is the sound pressure spectrum level at the hydrophone (dB re 1 µPa at 1 m),


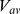
 is the average voltage output across 30 minutes of recorded signal (dB re 1 V),


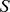
 is the system sensitivity of the sonobuoy (dB re 1 V µPa),


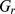
 is the gain due to receiver (dB),


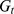
 is the gain due to the VHF transmitting and receiving system (dB re 1 V), and


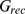
 is the gain due to the recording system (dB).

The mean sensitivity (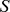) of the sonobuoys (Sparton Electronics AN/SSQ-57A) was -155.2 (range-158.5 to -151.0) dB re 1 V µPa depending on the frequency band ; the gain due to the receiver (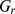)(AOR Ltd, AR 2001) was 50 dB; the gain due to the recording system (DAT TCD-D8) varied due to volume settings and the output from the recorder (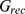) was measured in ^1^/_3_ octave frequency bands for each level of gain with respect to each frequency band.

The variance was estimated from the empirical variance of the Cue counts ( 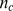 ) and Cue production rate (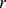), and variance of the estimated Distance from the hydrophones (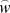) over 101 hydrophones (modified from [29], p. 78) as:
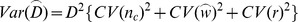
Where:


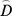
 is the estimated density,


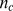
 is the number of detected cues,


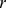
 is the cue production rate,


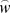
 is the estimated distance away from the hydrophones beyond which cues are assumed to not be detected.

**Age class of leopard seals.** – for each of the 101 sites where underwater recordings had been made within the pack ice (Figure 1B) an age classification was attributed according to the fundamental frequency of the calls within the 30 min recordings. As adult males tend to produce calls with higher peak fundamental frequencies [39], sites where calls tended to be above 313 Hz were assigned adult regions, and sites where calls tended to be below 313 Hz were assigned sub-adult regions. The sites were classified into one of three groups depending on the frequency of the calls: Mostly Adults, where more than 70% of calls were above 313 Hz; Mostly Sub-adults, where more than 70% of calls were below 313 Hz; and Both Adult and Sub-adults, where calls ranged across these frequencies.

**Visual surveys.** - Visual surveys were undertaken continuously during the daylight hours by four two-person teams on each side of the ship and from bridge and above-bridge positions. Further details are provided in [8].
